# Supplementary material for: The Effects of Temperature on Accident and Emergency Department Attendances in London: A Time-Series Regression Analysis
Source: Int J Environ Res Public Health. 2020 Mar 17;17(6):1957. doi: 10.3390/ijerph17061957 (PMC7142952; doi:10.3390/ijerph17061957)
Supplement: Supplementary file 1 [file ijerph-17-01957-s001.pdf]

International Journal of
Environmental Research and Public Health

MDPI

Supplementary Material

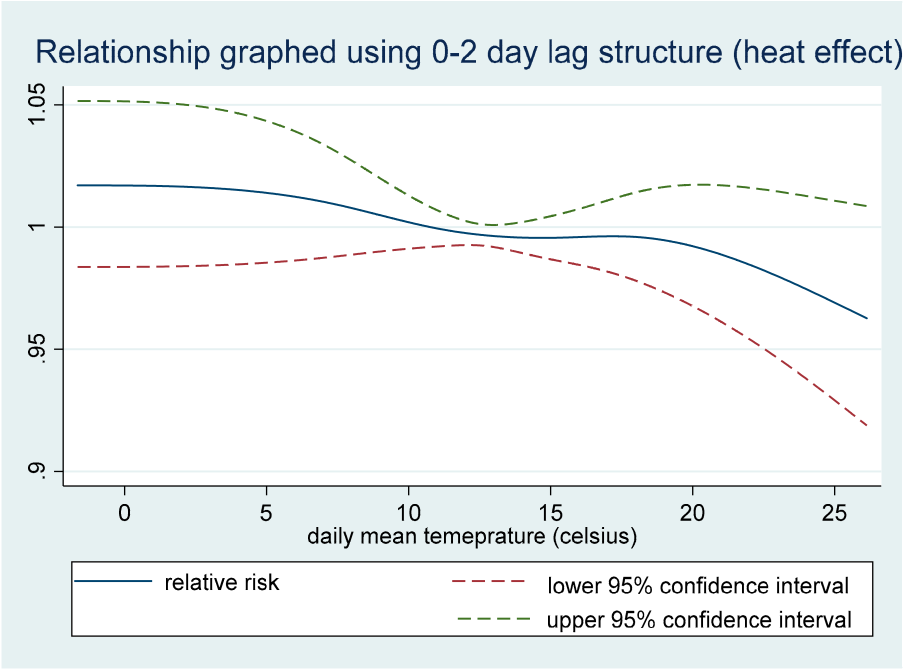

(a)

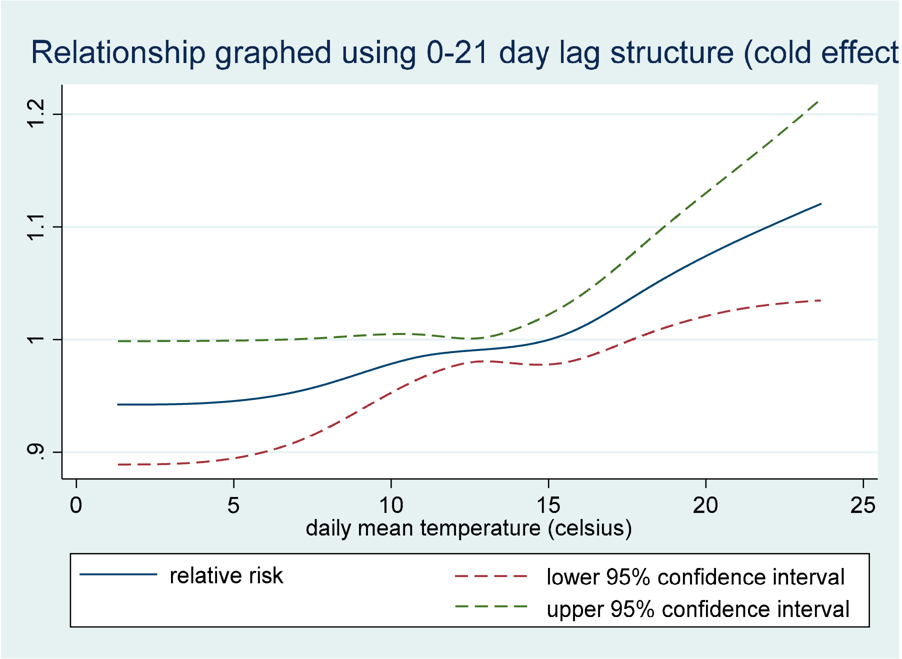

(b)

Figure S1. Relations between daily mean temperature and all A&E attendances, adjusted for seasonality, long term trend and day of the week. Temperature (˚C) on the x-axis **(a)** 0- to 2-day lag average and **(b)** 0- to 21-day lag average, and relative risk on the y-axis.

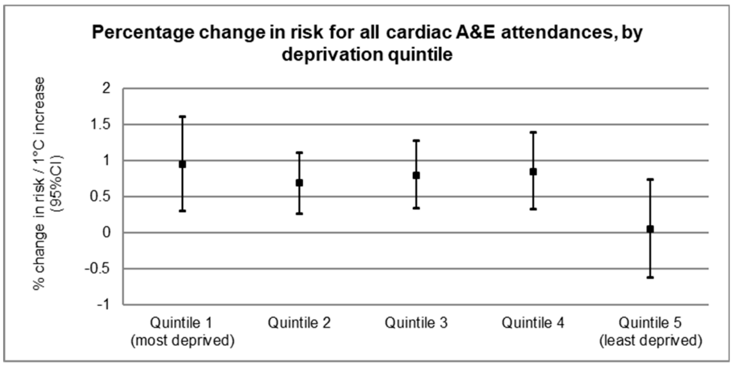
Percentage change in risk for all cardiac A&E attendances, by deprivation quintile
% change in risk / 1°C increase (95%CI)
2
1.5
1
0.5
0
-0.5
-1
Quintile 1 (most deprived)
Quintile 2
Quintile 3
Quintile 4
Quintile 5 (least deprived)

(a)

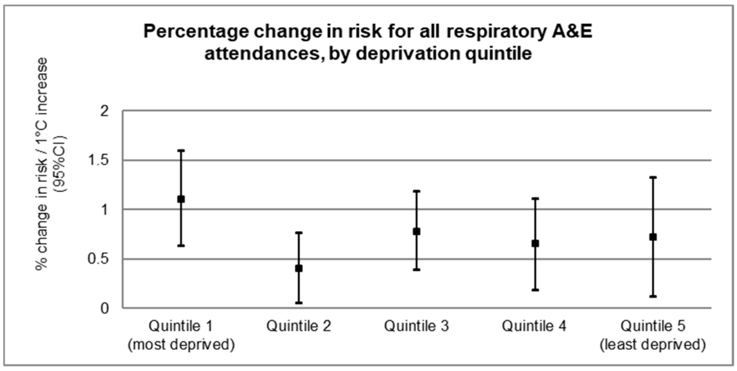
Percentage change in risk for all respiratory A&E attendances, by deprivation quintile
% change in risk / 1°C increase (95%CI)
2
1.5
1
0.5
0
Quintile 1 (most deprived)
Quintile 2
Quintile 3
Quintile 4
Quintile 5 (least deprived)

(b)

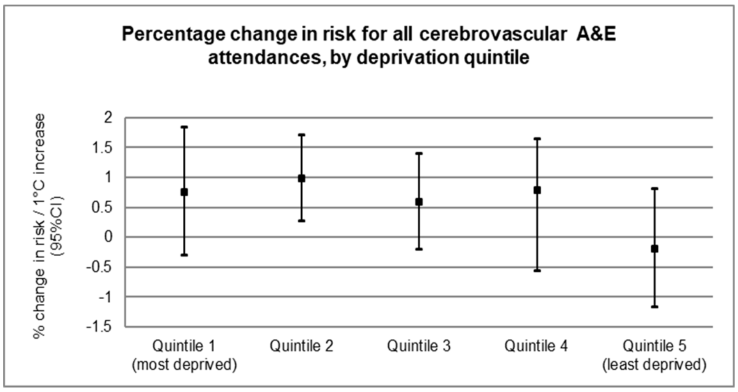
Percentage change in risk for all cerebrovascular A&E attendances, by deprivation quintile
% change in risk / 1°C increase (95%CI)
2
1.5
1
0.5
0
-0.5
-1
-1.5
Quintile 1 (most deprived)
Quintile 2
Quintile 3
Quintile 4
Quintile 5 (least deprived)

(c)

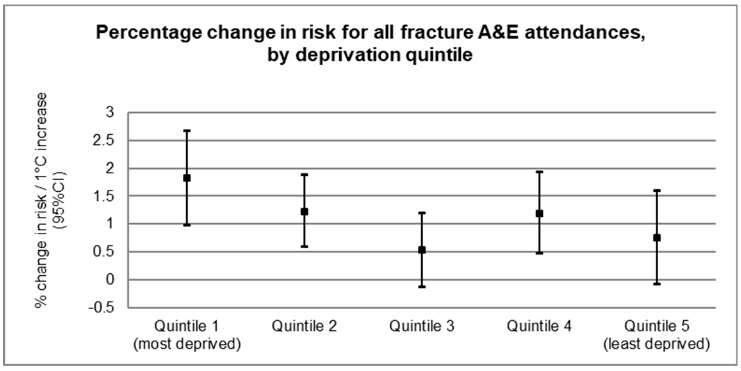
Percentage change in risk for all fracture A&E attendances, by deprivation quintile
% change in risk / 1°C increase (95%CI)
3
2.5
2
1.5
1
0.5
0
-0.5
Quintile 1 (most deprived)
Quintile 2
Quintile 3
Quintile 4
Quintile 5 (least deprived)

(d)

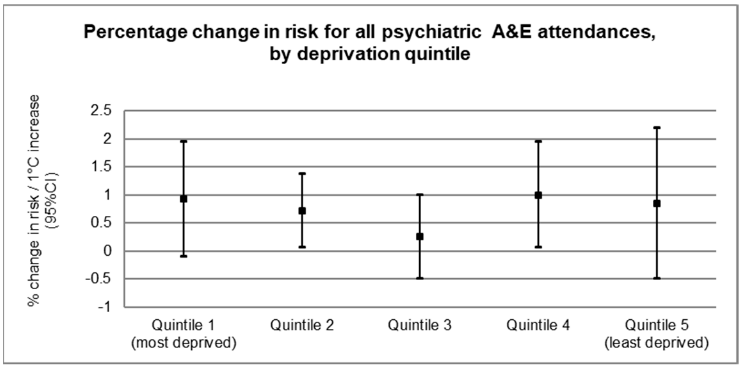

(e)

Figure S2. Effect modification by deprivation quintile for all cause-specific attendances: **(a)** cardiac, **(b)** respiratory, **(c)** cerebrovascular, **(d)** fractures and **(e)** psychiatric conditions.

Table S1. Summary of main findings from the regression model investigating the heat effect, by cause and by age.

HEAT RELATED PERCENTAGE CHANGE IN RISK OF ATTENDING A&E PER 1°C INCREASE IN MEAN TEMPERATURE												
	AT 0-DAY LAG			AT 1-DAY LAG			AT 2-DAY LAG			TOTAL EFFECT		
	%increase	95% CI	p-value	%increase	95% CI	p-value	%increase	95% CI	p-value	%increase	95% CI	p-value
**1) ALL A&E ATTENDANCES**												
**0-15 years**	0.7	(0.5,0.9)	<0.001	0.2	(-0.1,0.4)	0.145	0.5	(0.3,0.7)	<0.001	1.4	(1.2,1.5)	<0.001
**16-64 years**	0.4	(0.3,0.5)	<0.001	0.2	(0.1,0.3)	<0.001	0.2	(0.1,0.3)	<0.001	0.8	(0.7,0.9)	<0.001
**65-74 years**	0.4	(0.3,0.6)	<0.001	0.1	(-0.1,0.3)	0.234	0.1	(-0.1,0.3)	0.209	0.6	(0.5,0.8)	<0.001
**75-84 years**	0.5	(0.4,0.6)	<0.001	0.1	(-0.1,0.2)	0.756	0	(-0.1,0.3)	0.964	0.6	(0.4,0.7)	<0.001
**85+ years**	0.5	(0.4,0.7)	<0.001	0.2	(-0.1,0.4)	0.086	-0.1	(-0.1,0.1)	<.0.001	0.3	(0.2,0.5)	<0.001
**all ages**	0.5	(0.4,0.6)	<0.001	0.2	(0.1,0.3)	<0.001	0.2	(0.1,0.3)	<0.001	1.0	(0.8,1.4)	<0.001
**2) CARDIAC**												
**16-64 years**	0.5	(0.2,0.9)	0.003	-0.1	(-0.4,0.4)	0.886	0.3	(-0.1,0.6)	0.147	0.7	(0.4,1.1)	<0.001
**65-74 years**	0.7	(0.1,1.4)	0.026	0.1	(-0.7,0.9)	0.758	-0.1	(-0.10.4)	0.500	0.6	(0.1,1.3)	0.041
**75-84 years**	0.8	(0.2,1.4)	0.014	0.1	(-0.7,0.8)	0.849	-0.2	(-0.8,0.4)	0.580	0.7	(0.1,1.3)	0.023
**all ages**	0.7	(0.4,0.9)	<0.001	0.1	(-0.3,0.4)	0.790	-0.1	(-0.3,0.3)	0.948	0.7	(0.4,1.0)	<0.001
**3) RESPIRATORY**												
**0-15 years**	0.3	(-0.2,0.8)	0.194	0.1	(-0.4,0.7)	0.607	0.1	(-0.3,0.6)	0.606	0.6	(0.1,1.0)	0.009
**16-64 years**	0.3	(-0.1,0.7)	0.186	-0.2	(-0.8,0.3)	0.256	0.4	(-0.1,0.8)	0.067	0.4	(-0.1,0.7)	0.058
**65-74 years**	0.3	(-0.4,1.0)	0.392	0.7	(-0.1,1.5)	0.099	0.5	(-0.1,1.2)	0.112	1.5	(0.9,2.1)	<0.001
**75-84 years**	0.1	(-0.5,0.8)	0.744	0.6	(-0.2,1.3)	0.132	-0.1	(-0.7,0.6)	0.996	0.7	(0.1,1.3)	0.024
**all ages**	0.3	(0.1,0.6)	0.023	0.1	(-0.3,0.4)	0.530	0.3	(-0.1,0.5)	0.066	0.7	(0.4,0.9)	<0.001
**4) CEREBROVASCULAR**												
**16-64 years**	1.1	(0.5,1.7)	<0.001	-0.1	(-0.7,0.7)	0.907	-0.1	(-0.6,0.5)	0.831	1.0	(0.5,1.6)	<0.001
**all ages**	1.0	(0.6,1.5)	<0.001	0.2	(-0.3-0.8)	0.418	-0.6	(-1.0, -0,1)	0.006	0.6	(0.2,1.0)	0.003
**5) PSYCHIATRIC**												
**16-64 years**	0.1	(-0.4,0.5)	0.711	0.1	(-0.5,0.6)	0.895	0.6	(0.1-1.0)	0.010	0.7	(0.3,1.1)	0.001
**all ages**	0.2	(-0.2,0.6)	0.368	0.1	(-0.1,0.6)	0.702	0.4	(-0.1, -0.8)	0.006	0.7	(0.3,1.0)	0.001
**6) SOCIAL**												

**all ages**	-0.1	(-0.8,0.6)	0.774	0.3	(-0.5,1.2)	0.522	0.2	(-0.4,1.0)	0.505	0.4	(-0.3,1.1)	0.225

(1) Abbreviations: CI confidence interval.

(2) The unconstrained distributed lag model controls for seasonality and trend as well as day of week, public holidays, relative humidity and influenza.

(3) Cause-specific age subgroups that had less than 10 daily counts were not included in the analysis due to lack of power.

(4) Results from fracture-related attendances presented in separate table as model contained a heat threshold.

Table S2. Percentage change in risk by deprivation quintile and cause.

DEPRIVATION	CAUSE -SPECIFIC ED ATTENDANCES		
	%change	95% CI	*p*-Value
	OVERALL ATTENDANCES		
Quintile 1	1.02	(0.93,1.10)	<0.001
Quintile 2	0.86	(0.76,0.95)	<0.001
Quintile 3	0.78	(0.69,0.87)	<0.001
Quintile 4	0.77	(0.67,0.86)	<0.001
Quintile 5	0.74	(0.62,0.86)	<0.001
	CARDIAC ATTENDANCES		
Quintile 1	0.95	(0.30,1.60)	0.004
Quintile 2	0.69	(0.26,1.10)	0.002
Quintile 3	0.80	(0.33,1.27)	0.001
Quintile 4	0.85	(0.32,1.39)	0.002
Quintile 5	0.054	(-0.63,0.73)	0.878
	RESPIRATORY ATTENDANCES		
Quintile 1	1.11	(0.63,1.59)	<0.001
Quintile 2	0.41	(0.05,0.76)	0.027
Quintile 3	0.78	(0.39,1.18)	<0.001
Quintile 4	0.66	(0.18,1.11)	0.006
Quintile 5	0.72	(0.12,1.32)	0.018
	CEREBROVASCULAR ATTENDANCES		
Quintile 1	0.76	(-0.31,1.83)	0.162
Quintile 2	0.98	(0.26,1.70)	0.008
Quintile 3	0.59	(-0.2, -1.39)	0.147
Quintile 4	0.79	(-0.57,1.63)	0.06
Quintile 5	-0.19	(-1.18,0.81)	0.709
	FRACTURE ATTENDANCES *		
Quintile 1	1.82	(0.97,2.66)	<0.001
Quintile 2	1.23	(0.59,1.88)	<0.001
Quintile 3	0.53	(-0.13,1.19)	0.115
Quintile 4	1.19	(0.47,1.93)	0.001
Quintile 5	0.76	(-0.09,1.60)	0.081
	PSYCHIATRIC ATTENDANCES		
Quintile 1	0.92	(-0.10,1.94)	0.078
Quintile 2	0.72	(0.06,1.37)	0.033
Quintile 3	0.26	(-0.49,1.00)	0.494
Quintile 4	1.00	(0.06,1.94)	0.037
Quintile 5	0.85	(-0.49,2.19)	0.217

(1) Abbreviations: CI confidence interval .

(2) Quintile 1: most deprived; Quintile 5: least deprived .

(3) Percentage change in heat-related risk for attending A&E for every 1 °C change in mean temperature, adjusted for cold effects, seasonality and long-term trends, day of the week, relative humidity and weekly influenza counts.

(*) The regression model for fractures includes a heat threshold of 16 °C; therefore, the results shown are the percent change in risk for every 1 °C increase in mean temperature above 16 °C.
